# Lattice defects in quinacridone

**DOI:** 10.1107/S205252062200779X

**Published:** 2022-09-09

**Authors:** Dominik Brey, Barbara Scherer, Martin U. Schmidt

**Affiliations:** a Goethe-Universität, Institut für Anorganische und Analytische Chemie, Max-von-Laue-Str. 7, 60438 Frankfurt am Main, Germany; b Goethe-Universität, Institut für Physikalische und Theoretische Chemie, Max-von-Laue-Str. 7, 60438 Frankfurt am Main, Germany; University of Geneva, Switzerland

**Keywords:** lattice defects, crystal modelling, orientation fault, twinning, organic semiconductor, commensurate misfit-layer structure

## Abstract

Various lattice defects in the α^I^-phase of quinacridone (C_20_H_12_N_2_O_2_) were simulated using lattice-energy minimizations. α^I^-Quinacridone forms a chain structure in *P* 1, *Z* = 1. The molecules are connected by hydrogen bonds along [010], by π-stacking along [100] and by weak van der Waals interactions along [001]. α^I^-Quinacridone is inherently nanocrystalline. Lattice defects were calculated in correspondingly large supercells with up to 4464 atoms, using a previously evaluated force field. Vacancies, vacancy aggregates and interstitial molecules are energetically very unfavourable. A misorientation of a single molecule (flip around [010] by 180°) causes an energy increase of 243.7 kJ mol^−1^. Various edge and screw dislocations were investigated. A screw dislocation along [010] causes an energy increase of Δ*E* = 38.0 kJ mol^−1^ per molecule, all other line dislocations are even worse. In contrast, the rotation of an entire chain around the chain axis [010] by 180° leads to only a very small energy increase (Δ*E* = 1.6 kJ mol^−1^) and the real crystals probably contain a high number of such defects. Various planar defects were calculated, including different stacking disorders and misfit-layer structures with two different types of layers having different lateral periodicities. Stacking faults along [001] with herringbone stacking instead of parallel stacking are energetically quite favourable (Δ*E* = 2.2 kJ mol^−1^); the same is true for domains with misoriented molecules in the [001] direction. As an example for a bulk defect, domains are calculated in which blocks of 4 × 4 chains are rotated by 180° around [010], which leads to an energy increase of only 1.1 kJ mol^−1^. Twinning by mirroring at the (001) plane is energetically favourable (Δ*E* = 0.9 kJ mol^−1^). This twinning was observed in an HRTEM image. It is probable that the crystallites also contain rotations of chains, layers or blocks around [010] by 180°, but these defects cause only a very slight modification of the molecular packing, which was not observable in the HRTEM image. The lattice defects in α^I^-quinacridone investigated here provide an insight into lattice defects, their energies and local structures. Similar lattice defects are expected to occur also in other similar organic chain structures.

## Abstract 2

Various lattice defects in the α^I^-phase of quinacridone (C_20_H_12_N_2_O_2_) were simulated using lattice-energy minimizations, including vacancies, stacking faults, screw and edge dislocations, twinning and orientational faults. Twinning and orientational faults of entire chains were calculated to occur most frequently.

## 1. Introduction

**Figure 1:**
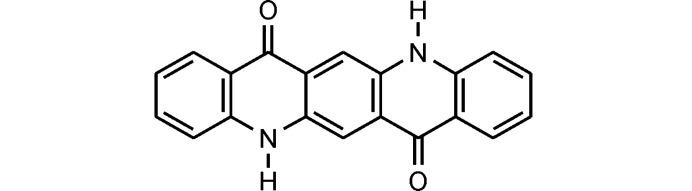
Chemical diagram for quinacridone.

Quinacridone (Fig. 1 ▸) is an organic semiconductor. Organic semiconductors are used for electronic devices such as organic light-emitting diodes, organic solar cells (organic photovoltaic) and organic field-effect transistors. The electronic properties of semiconductors strongly depend on the type and frequency of lattice defects, such as dislocation lines or impurities. The nature, local structure and frequency of lattice defects in inorganic semiconductors, *e.g.* silicon, has been widely studied for many decades (see *e.g.* Kolbesen & Cerva, 1999 ▸; Kolbesen, 2014 ▸). For organic semiconductors, much less work has been reported, hitherto (see *e.g.* Sherwood, 1969 ▸; Desvergne *et al.*, 1974 ▸; Mokichev & Pakhomov, 1982 ▸; Ide *et al.*, 1993 ▸; Cuppen *et al.*, 2004 ▸; Kang *et al.*, 2005 ▸; Walker *et al.*, 2005 ▸; Chapman *et al.*, 2006 ▸; Wu *et al.*, 2017 ▸; Li *et al.*, 2022 ▸).

Typical lattice defects in inorganic solids are: point defects, *e.g.* vacancies, interstitial atoms, or dopant atoms; line defects, *e.g.* edge or screw dislocations; planar defects such as stacking faults or small-angle grain boundaries; and bulk defects such as voids. All these defects may occur in organic crystals, too (see *e.g.* Scheffen-Lauenroth *et al.*, 1981 ▸; Olson *et al.*, 2018 ▸). Additionally, there can be orientational defects (or orientational disorder) of side groups, or of entire molecules, *e.g.* head-to-tail disorder. The orientational defect may be located in single molecules, or can proliferate to chains or layers of molecules in an incorrect orientation. All these lattice defects were investigated in this work. Additionally, organic crystals can incorporate foreign molecules such as impurities, byproducts, decomposition products, water and solvent molecules which will be the subjects of further work.

As a model compound, we chose the α^I^-phase of quinacridone. Quinacridone (Fig. 1 ▸) is an industrial organic pigment used for the colouration of paints, coatings, plastics and printing inks (Hunger & Schmidt, 2018 ▸). In the International Colour Index, it is registered as Pigment Violet 19 (Colour Index International, 2022 ▸; Abel, 1998 ▸). There are four polymorphic forms. The α^I^- and α^II^-phases result from the synthesis of the compound, depending on the synthetic conditions. Heating the α^I^-phase in NaOH yields the β-phase, whereas heating in organic solvents, *e.g.* iso­butanol, results in the γ-phase. The crystal structure of the α^I^-phase was determined by crystal structure prediction followed by Rietveld refinement (Leusen, 1994 ▸, 1996 ▸; Paulus *et al.*, 2007 ▸). The structures of the β- and γ-phases were determined by single-crystal X-ray diffraction (Paulus *et al.*, 1989 ▸; Potts *et al.*, 1994 ▸; Mizuguchi, Sasaki & Tojo, 2002 ▸; Nishimura *et al.*, 2006 ▸).

The α^II^-phase (at that time there was no distinction between the phases α^I^ and α^II^; both were called α-phase) was investigated by Lincke & Finzel (1996 ▸), using a limited-quality powder pattern of the α^II^-phase. They constructed a structure in 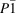 with two independent molecules per unit cell, both located on inversion centres. The molecules were assumed to form a criss-cross pattern like in γ-quinacridone. This packing was manually fitted to the powder pattern (Lincke & Finzel, 1996 ▸). However, the final fit was not convincing, and the structure remained questionable (Paulus *et al.*, 2007 ▸). Finally, the structure of the α^II^-phase was determined by electron diffraction (Gorelik *et al.*, 2016 ▸), revealing a chain structure and, thereby, disproving the structural model of Lincke & Finzel (1996 ▸).

The α^I^-phase crystallizes in 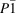 with *Z* = 1, all other phases crystallize in *P*2_1_/*c*, *Z* = 2.

**Figure 2:**
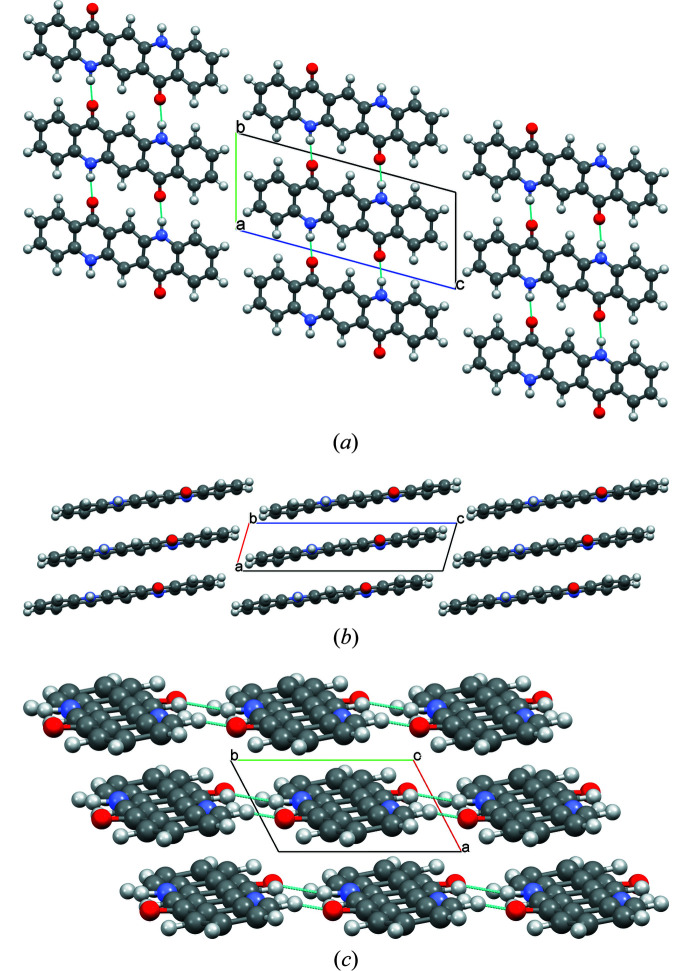
Crystal structure of α^I^-quinacridone (without lattice defects). View directions (*a*) [100], (*b*) [010], (*c*) [001]. Colour code for all figures: C grey, H white, N blue, O red. Hydrogen bonds are shown as dashed lines. Note that in this work a different unit-cell setting is used than in earlier work (for details, see Section 2.1). The structure after unit-cell transformation and lattice-energy optimization is shown.

**Figure 3:**
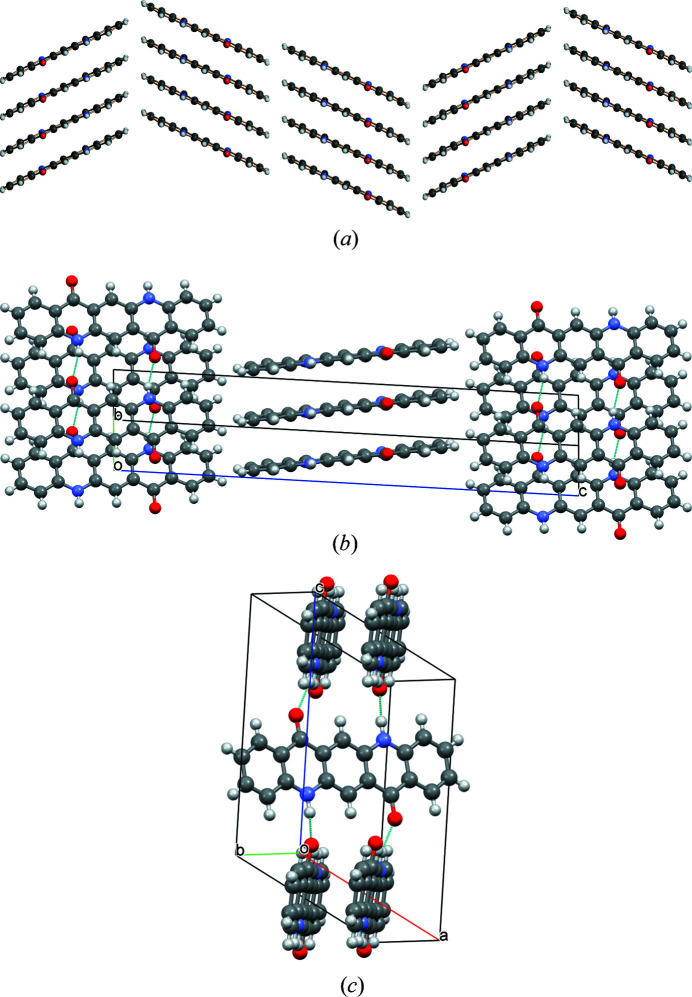
Crystal structures of other polymorphs of quinacridone: (*a*) α^II^-phase (with stacking disorder), (*b*) β-phase and (*c*) γ-phase.

The quinacridone molecules are always planar or close to planar. In all phases the molecules are situated on crystallographic inversion centres. In the α^I^-, α^II^- and β-phases, each molecule is connected to two neighbouring molecules by two hydrogen bonds each, resulting in chains. In the α^I^-phase all chains are parallel (Fig. 2 ▸). The chains are not fully planar, but exhibit steps of approximately 0.6 Å between the molecules [Fig. 2 ▸(*c*)]. In the α^II^-phase the chains form, in principle, a herringbone packing; but actually the α^II^-phase exhibits a stacking disorder with a mixture of herringbone and parallel arrangements of neighbouring sheets, as revealed by electron diffraction and lattice-energy minimizations [Fig. 3 ▸(*a*); Gorelik *et al.*, 2016 ▸]. In the β-phase the chains extend in two different directions, [110] and 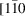 ], which form an angle of 69° [Fig. 3 ▸(*b*)]. In the three phases, the chains are stacked on top of each other, resulting in layers, but the stacking of the layers is different. In the α^I^-phase, neighbouring layers are translationally equivalent, in the β-phase they are rotated by 69° and in the α^II^-phase by 180°. Correspondingly, these three structures belong to the same polytype family. In the γ-phase, each molecule is connected to four neighbouring molecules, resulting in a criss-cross pattern [Fig. 3 ▸(*c*)]. In all phases, the molecules are densely packed. The densities are high (α^I^ = 1493 kg m^−3^, α^II^ = 1414 kg m^−3^, β = 1540 kg m^−3^, γ = 1470 kg m^−3^). The lattice energies are high, and all phases are fully insoluble in water and all solvents at ambient conditions. Quinacridone is thermally stable in air to temperatures of about 600–700 K, when it starts to sublime.

The crystal structures of quinacridone have a strong effect on the electronic structure, as is evident from the different colours of the individual polymorphs. The α^I^-phase has a dull reddish-violet shade, the α^II^-phase is red, the β-phase is reddish violet and the γ-phase is red, too (but slightly more yellowish than α^II^-quinacridone). In solution, quinacridone is yellow to orange. A similar colour shift between solution and solid state is also observed for several other organic pigments used in car coatings, including diketo­pyrrolo­pyrrole or perylene pigments. In all these cases, the observed shift from a yellow to orange colour in solution to a red or violet in the solid state is caused by the stacking of the molecules, resulting in a classic blue-shifted H-aggregate. Thus, the red colour of most red cars is only a solid-state effect; without the intermolecular interactions of the molecules in the crystals, the cars would be yellow or orange.

The α^I^-phase of quinacridone presents itself as an exemplary model system for the investigation of lattice defects, because the molecule is rigid and the crystal structure is quite simple. The unit cell contains only one molecule, which is situated on a crystallographic inversion centre. Similar crystal structures in 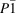 , *Z* = 1, also consisting of planar molecules connected by double hydrogen bonds into parallel chains, have also been reported for other organic semiconductors, *e.g.* 2,9-di­methyl­quinacridone (Pigment Red 122; Mizuguchi, Senju & Sakai, 2002 ▸) and diketo­pyrrolo­pyrrole (Pigment Red 255; Mizuguchi *et al.*, 1992 ▸).

There is a further advantage to using the α^I^-phase as a model system. The molecules are connected in the **a** direction by π-stacking, in the **b** direction by hydrogen bonds and in the **c** direction by van der Waals interactions between C and H atoms. Hence, the effect of the different intermolecular interactions on the lattice defects can clearly be distinguished.

Industrially, the α^I^-phase is obtained as follows. The starting material, 2,5-bis­(phenylamino)terephthalic acid is heated in molten polyphospho­ric acid to 100–150°C. The resulting dark-blue mixture of (probably protonated) quinacridone in hot polyphospho­ric acid is poured into ice-water. The α^I^-quinacridone immediately precipitates as a nanocrystalline powder. The α^I^-phase is always nanocrystalline. Recrystallization is not possible because of the insolubility in all media. Treatment with solvents in suspension at elevated temperature either does not improve the crystallinity, or results in a phase transformation into the more stable β- or γ-phases. The best powder which we ever obtained had a domain size of 20 nm, as determined by Rietveld refinement. Samples from industrial production may be close to amorphous. Correspondingly, the α^I^-phase certainly contains a high number of lattice defects. However, nothing is known of the nature of these defects, their local structure or their frequency. Experimental investigations on the defects in this nanocrystalline powder would be difficult. In contrast, the simulation of the lattice defects by lattice-energy minimizations is feasible.

For the simulation of the lattice defects, correspondingly large superstructures are constructed, *e.g.* 5 × 5 × 5 unit cells for a point defect such as a vacancy. Such large supercells at present prevent the minimization of the lattice energy with quantum-mechanical methods. Hence, we used force-field methods, which were already successfully employed for the calculation of the crystal structures of α^I^-quinacridone (Leusen, 1994 ▸, 1996 ▸) and 2-methyl-quinacridone (Schlesinger *et al.*, 2020 ▸).

In this paper, the applied force field is evaluated first. Subsequently, various types of point defects, line defects, planar defects and bulk defects of α^I^-quinacridone are investigated. Finally, we compare the simulation results with an experimental HRTEM image of α^I^-quinacridone.

## 2. Calculation details and experimental details

### 2.1. Force fields

Two different force fields were tested: the Dreiding force field (Mayo *et al.*, 1990 ▸) and a tailor-made force field fitted to lattice-energy calculations by dispersion-corrected density-functional theory (DFT-D), as described by Neumann *et al.* (2008 ▸). For the atomic charges, various approaches were tested: (1) the Gasteiger method (Gasteiger & Marsilli, 1980 ▸); (2) the charge-equilibrium method as implemented in *Materials Studio* (Accelrys, 2008 ▸); (3) charges calculated by the electrostatic potential (ESP) method, based on HF/6-31G** calculations and (4) charges associated with the tailor-made force-field. Different algorithms for the summation of the electrostatic energy were employed: atom-based, group-based and Ewald summation.

Finally, all lattice defects were calculated with the Dreiding force field, ESP charges and Ewald summation for Coulomb and van der Waals interactions.

DFT-D calculations of the four polymorphs of quinacridone without lattice defects were performed with the program *CASTEP* (Clark *et al.*, 2005 ▸), with the PBE functional (Perdew *et al.*, 1996 ▸) and a dispersion correction of Grimme (2006 ▸). The unit-cell parameters were optimized together with all atomic coordinates.

### 2.2. Programs

The best program for crystal modelling studies on organic compounds with force fields was, in our opinion, *Cerius^2^* (Accelrys, 2005 ▸). However, *Cerius^2^* requires an SGI workstation with IRIX operating system, which is outdated. Thus, we used its successor program, *Materials Studio* (Version 4.4, Accelrys, 2008 ▸), which runs on a Windows PC. Drawings were created with *Mercury* (Macrae *et al.*, 2020 ▸) and *SCHAKAL* (Keller, 1999 ▸).

### 2.3. Model building and optimization

α^I^-Quinacridone crystallizes in 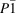 , *Z* = 1, with unit-cell parameters at room temperature of *a* = 3.8017 (15), *b* = 6.612 (3), *c* = 14.485 (6) Å, α = 100.68 (8), β = 94.40 (6), γ = 102.11 (5)°, *V* = 346.67 (11) Å^3^ (Paulus *et al.*, 2007 ▸). For the simulation of lattice defects, the unit cell was transformed with ***a*** ′ = −***a***, ***b*** ′ = ***a*** + ***b***, ***c*** ′ = −***b*** − ***c*** and the molecules were shifted by (½, 0, ½). In the resulting unit-cell setting, the *a* axis corresponds to the thickness of the molecule, the *b* axis to the width and the *c* axis to the length of the molecule. The molecule is situated on a crystallographic inversion centre at (½, ½, ½) in the centre of the unit cell. This setting facilitates the construction, evaluation and description of the various lattice defects.

For the construction of the lattice defects, correspondingly large supercells were set up. For example, for point defects such as vacancies we used a 3 × 3 × 3 or 5 × 5 × 5 supercell, for line defects such as edge or screw dislocations in the **c** direction a 9 × 6 × 1 supercell, and for planar defects in (100) direction a 8 × 1 × 1 supercell. Subsequently, the molecules were manually removed, rotated or shifted, to obtain a sensible starting point for the following lattice-energy minimizations. In complicated cases, such as screw or edge dislocations, several different models were set up and optimized, in order to find the energetically most favourable local structure for a given defect type. Screw and edge dislocations as well as twinning violate 3D periodicity of the crystal. To counteract this effect, we always employed them pairwise.

Exemplary manually constructed starting structures along with their optimized versions are shown in Sections 4.1, 5.2.3, 5.3.1 and 5.3.2.

For the optimization, the convergence criteria in *Materials Studio* were set to ‘ultrafine’. Calculation times were in the order of 2 min to 2 h on a standard PC.

For the calculation of point and line defects, the unit-cell parameters were kept fixed to the values of the optimized, undisturbed structure. This procedure reflects the assumption that the overall unit-cell parameters of a crystal do not change upon the occurrence of a point or line defect. Additional calculations were performed with variable unit-cell parameters. If the supercell is not too small, these calculations yielded very similar structures and energies as those with fixed unit-cell parameters. In contrast, calculations of planar defects such as stacking faults require the corresponding unit-cell parameters to be optimized. For example, a planar defect in the (100) plane requires *a*, β and γ to be optimized, whereas α, *b* and *c* can be fixed. Volume defects of limited size were treated like point and line defects, *i.e.* with fixed unit-cell parameters.

The optimized, undisturbed structure of α^I^-quinacridone has a total force-field energy of −309.264 kcal mol^−1^ = −1294.52 kJ mol^−1^. Test calculations proved that the structure and energy are independent of the unit-cell setting. This energy value served as reference for all lattice defects. The relative energies of the various lattice defects described in this paper are given in kJ mol^−1^ per molecule per lattice defect (1 kcal = 4.184 kJ), unless stated otherwise. For example, the energy of a vacancy in a 5 × 5 × 5 supercell is calculated as the difference between the total energy of the optimized supercell and 5^3^ − 1 times the lattice energy of the optimized initial cell with *Z* = 1. For line defects and planar defects, the energy is given per unit-cell lengths of the undisturbed structure in the corresponding directions, *i.e.* per molecule of the lattice defect.

For most lattice defects, the lattice energies of the optimized structures depend only slightly on the size of the unit cell. For example, the energy of a vacancy is 231.69 kJ mol^−1^ in a 3 × 3 × 3 supercell and 233.37 kJ mol^−1^ in a 5 × 5 × 5 supercell. The energy of the 3 × 3 × 3 supercell model is reduced to 230.66 kJ mol^−1^, if the unit-cell parameters are optimized too.

### 2.4. Pair-distribution function analyses

X-ray powder diffraction data were recorded at 6ID-D MUCAT beamline at the Advanced Photon Source (APS) at Argonne National Laboratory, using a wavelength of 0.1428 Å. The sample was prepared on Kapton tapes and measured at 100 K. The pair distribution function (PDF) was calculated with *PDFgetX2* (Qiu *et al.*, 2004 ▸), using a *Q*_max_ of 21 Å^−1^.

## 3. Force-field validation

The force field was evaluated by lattice-energy minimization of all four polymorphs of quinacridone, using different force fields, different atomic charges and different methods for the summation of the electrostatic energy. The best results were obtained for the Dreiding force field in combination with *ab* *initio* ESP charges and Ewald summation. Upon optimization, the crystal structures changed only slightly (Tables 1 ▸ and S2 in supporting information). The resulting lattice energies agree with the experimental observation that the β- and γ-phases are thermodynamically more stable than the α-phases (see Table S3). (Kinetically, all phases are stable at ambient conditions for decades.) This force field was used for the calculation of all lattice defects.

Interestingly, exactly this combination of a Dreiding force field, 6-31G**-ESP charges and Ewald summation was already successfully used in 1994 for the crystal structure prediction of quinacridone (Leusen, 1994 ▸, 1996 ▸), which led to the crystal structure determination of α^I^-quinacridone.

The optimized crystal structure of α^I^-quinacridone is shown in Fig. 2 ▸. The unit-cell parameters are given in Table 1 ▸. Henceforth, these unit-cell parameters are denoted as *a*_0_, *b*_0_, *c*_0_, α_0_, β_0_ and γ_0_. This structure served as the basis for the construction and lattice-energy minimizations of all lattice defects.

## 4. Results and discussion: lattice defects: point defects

### 4.1. Vacancies

Vacancies are frequently found in metals, *e.g.* silicon, or in ionic solids, *e.g.* metal halides, where they show up as missing atoms or ions. In molecular crystals, a vacancy corresponds to a missing entire molecule.

**Figure 4:**
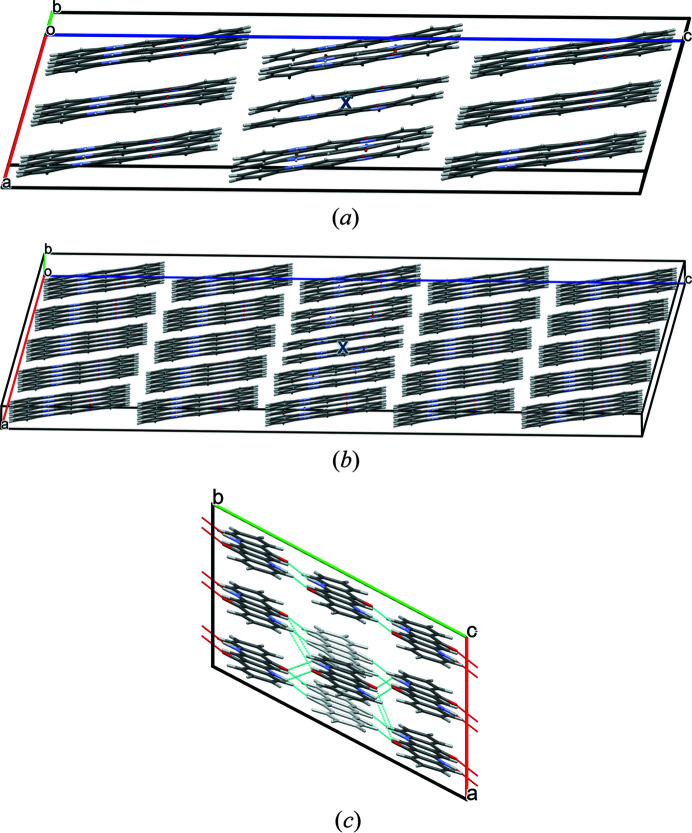
Vacancy. (*a*) Superstructure with 3 × 3 × 3 unit cells after optimization. Perspective view, approximately along [010]. The vacancy is marked by x. (*b*) Superstructure with 5 × 5 × 5 unit cells, containing 4464 atoms, after optimization. View direction approximately [010]. (*c*) Starting structure with a molecule in the centre of the vacancy, before optimization. In (*c*) the layer with the vacancy is shown in dark colours, the other layers are light grey.

For α^I^-quinacridone, vacancies were calculated with a supercell of 3 × 3 × 3 or 5 × 5 × 5 unit cells. The latter one contains 5^3^ − 1 = 124 molecules with 4464 atoms. The resulting optimized structure is shown in Figs. 4 ▸(*a*) and 4 ▸(*b*). Astonishingly, the molecules in the neighbourhood of the vacancy show no tendency to move towards the empty space. The molecular positions change by less than 0.1 Å. The reason might be that all neighbouring molecules are fixed to their neighbours by hydrogen bonds and π-stacking. The molecular packing of α^I^-quinacridone is very space-efficient, and very favourable in terms of intermolecular energies. Hence, every molecular movement by more than 0.1 Å would cause these intermolecular interactions to deteriorate.

The vacancy leads to a very high energy increase of 233.4 kJ mol^−1^. This energy increase is caused by the loss of two hydrogen bonds (25.5 kJ mol^−1^), but even more by the loss of the intermolecular van der Waals energy (154.2 kJ mol^−1^) and the electrostatic energy (66.4 kJ mol^−1^).

We also investigated the instance in which one of the neighbouring molecules is positioned in the centre of the vacancy, so that the H atoms of all NH groups are involved in hydrogen bonds, some of them being bifurcated [see Fig. 4 ▸(*c*)]. However, their geometry strongly deviates from usual hydrogen-bond geometries. Apparently, this arrangement is energetically unfavourable: upon optimization the central molecule moves back to its original position, and the refinement converges to the situation with a full vacancy in an otherwise undisturbed structure as shown in Fig. 4 ▸(*a*).

The high energy reveals that such an empty vacancy is very unlikely. Under real experimental conditions, the vacancies are probably filled with other molecules, such as water, solvents or byproduct molecules.

### 4.2. Vacancy aggregates

Vacancy aggregates, *e.g.* two of three missing neighbouring molecules, are energetically very unfavourable, too.

### 4.3. Interstitial molecules

**Figure 5:**
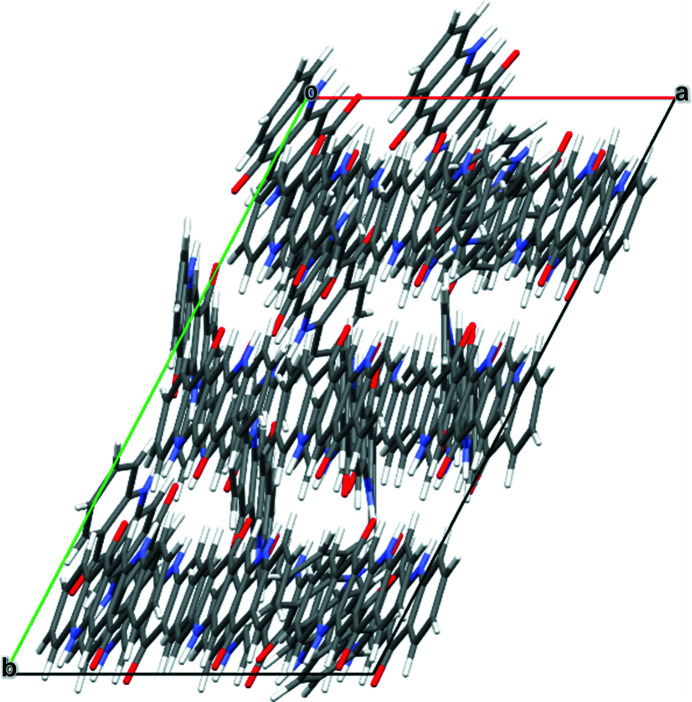
Attempt to calculate a structure with one interstitial molecule. This figure and all following figures show the structures after optimization. The additional molecule leads to a complete distortion of the whole structure in its vicinity.

α^I^-Quinacridone has an efficient molecular packing with a density of 1493 kg m^−3^. Any attempt to squeeze an additional molecule into this structure leads to strong local distortions and a high energy increase. An example with an energy increase of about 670 kJ mol^−1^ is shown in Fig. 5 ▸.

### 4.4. Orientation faults

**Figure 6:**
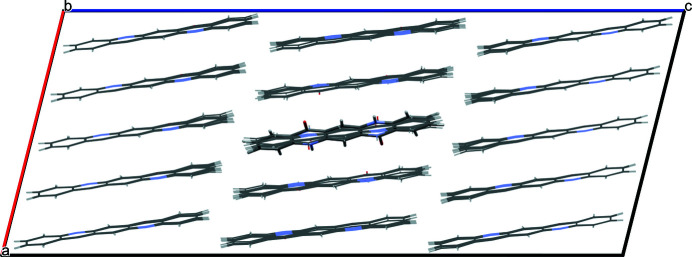
Orientation fault of a single molecule.

A rotation of a molecule by 180° around its long molecular axis or its medium molecular axis results in a mutual exchange of CO and NH groups. Such an orientational ‘head-to-tail disorder’ of a single molecule is sterically quite possible, but results in energetically unfavourable C=O···O=C and N—H···H—N contacts. The misoriented molecule evades these contacts by rotating out of the plane by 11.80°, see Fig. 6 ▸. The energy is +243.7 kJ mol^−1^.

### 4.5. Combination of orientation fault and vacancy

**Figure 7:**
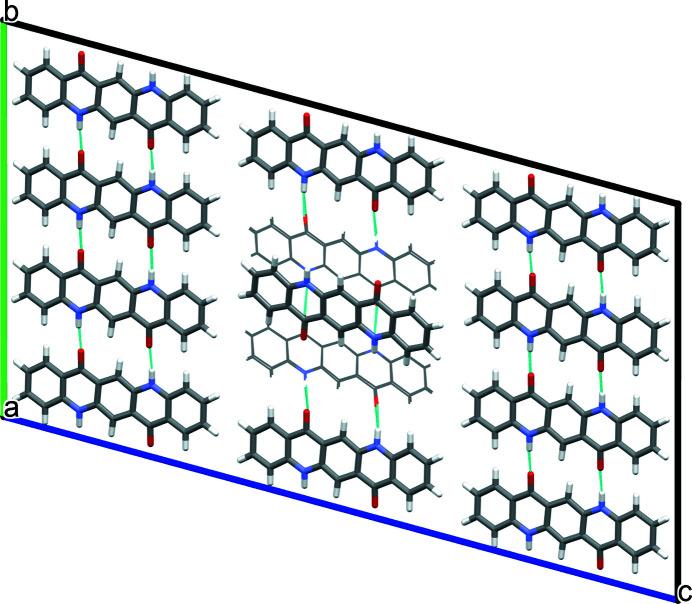
Combination of orientation fault and vacancy. The central layer is shown as bold sticks, the other layers in wireframe style.

In order to provide more space for the misoriented molecule, we tested the combination of an orientation fault with a neighbouring vacancy. The misoriented molecule was placed in the centre of the available space and the structure was optimized. The resulting structure is shown in Fig. 7 ▸. The energy of Δ*E* = 366.4 kJ mol^−1^ is lower than the sum of the structures with a vacancy and with a misoriented molecule, but still higher than for a misoriented molecule without a vacancy. Hence, a misoriented single molecule will be extremely rare in a real crystal.

## 5. Line defects

Line defects can occur in all spatial directions. For α^I^-quin­acridone the principal directions are:

(*a*) [100]: π-stacking of the molecules,

(*b*) [010]: hydrogen bonds,

(*c*) [001]: weak van der Waals interactions between the ends of the molecules.

**Figure 8:**
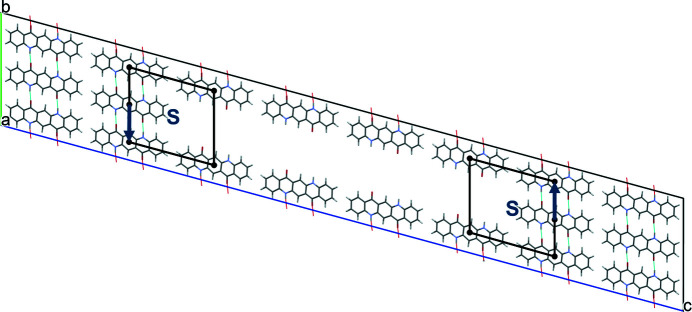
Edge dislocations along [100] with Burgers vectors (0,1,0) and 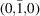 . The two dislocation lines run in the view direction of [100] and are marked by S. The two ‘circuits around the dislocation line’ are shown in black; the Burgers vectors are shown in blue.

Edge and screw dislocations cause a violation of the translational periodicity in their vicinity. Thus, a single edge or screw dislocation cannot be handled with a three-dimensionally periodic supercell. Therefore, all our models contain a pair of edge or screw dislocations so that the sum of the two Burgers vectors vanishes (see Fig. 8 ▸). The resulting pair of parallel dislocation lines can be regarded as a section of a dislocation loop. This approach spawns the problem that the local structure and the energy of edge and screw dislocations depend not only on the direction of the dislocation line and the Burgers vector, but also on the relative position and distance of the two dislocation lines in the supercell. However, a combination of two dislocation lines may well be present in a real crystal.

Various models for edge and screw dislocations were tested. In the following we focus on those models which remained chemically sensible after optimization.

The total energy of a line defect depends on the length of the dislocation line in the crystal. In our calculations, the dislocation line is assumed to be infinite, and the models are translationally periodic in the direction of the dislocation line. In the following, the energies are given per dislocation line per translational period, *i.e.* per unit-cell parameter of the unit cell in the corresponding direction, *e.g.* per 6.386 Å in the [010] direction.

Apart from edge and screw dislocations, two other types of line dislocations were investigated: missing molecular chains and chains with incorrect molecular orientations.

### 5.1. Line vacancy along [010] (missing molecular chain)

**Figure 9:**
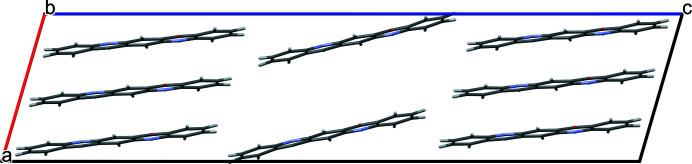
Line vacancy after optimization with fixed unit-cell parameters.

In the [010] direction, the molecules are connected by hydrogen bonds. Removing one entire chain along [010] does not break any hydrogen bonds, but the van der Waals interactions to molecules in neighbouring chains are lost. The neighbouring chains only show a slight tendency to fill the empty space (see Fig. 9 ▸). The energy increases by 145.0 kJ mol^−1^ per missing molecule. As for a vacancy and a point defect, in a real crystal, the empty space would be filled by other molecules.

Line vacancies along other spatial directions would cause the breaking of many hydrogen bonds and are energetically very unfavourable too.

### 5.2. Edge dislocations and dislocation loops

In edge dislocations, the Burgers vector is (nearly) perpendicular to the direction of the dislocation line. We investigated dislocations along [100], [010] and [001], with Burgers vectors (0,1,0), (0,0,1) and (1,0,0).

#### 5.2.1. Dislocation line parallel to [100] and Burgers vector (0,1,0)

A model with two edge dislocations, amounting to a dislocation loop, was assembled by constructing a supercell of 1 × 3 × 8 unit cells and removing four consecutive molecules along [001]. The remaining molecules show no tendency to move into the vacancy, because they are held in place by hydrogen bonds (see Fig. 8 ▸). As such, a relatively large vacancy remains, which is energetically unfavourable at 295.0 kJ mol^−1^ per dislocation line.

#### 5.2.2. Dislocation line parallel to [100] and Burgers vector (0,0,1)

**Figure 10:**
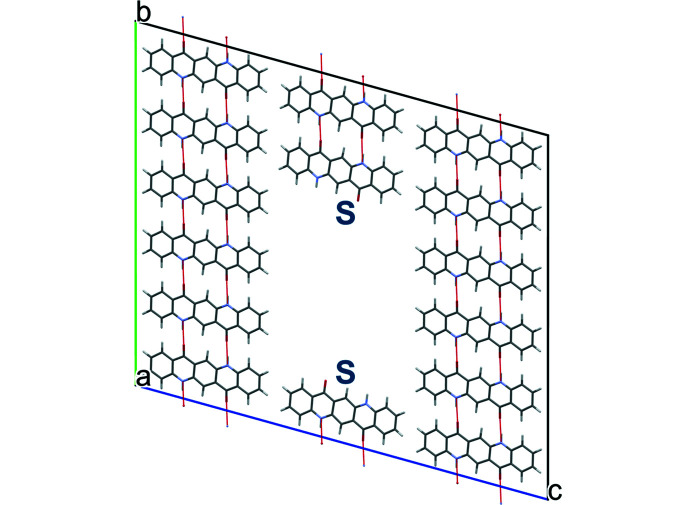
Edge dislocation along [100] with the Burgers vector (0,0,1). View direction [100]. The positions of the dislocation lines are marked by S.

The dislocation line was generated in a similar fashion as the one before, by construction of a supercell of 1 × 6 × 3 unit cells and the removal of three molecules along [010] (see Fig. 10 ▸). As before, a large vacancy persists because the remaining molecules are fixed in place by hydrogen bonds, resulting in an energy of 104.1 kJ mol^−1^.

#### 5.2.3. Dislocation line parallel to [010] and Burgers vector (1,0,0)

**Figure 11:**
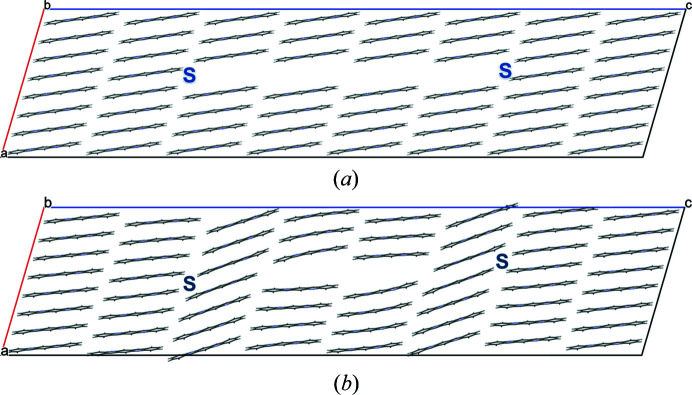
Edge dislocation along [010] and Burgers vector (1,0,0). (*a*) Starting structure and (*b*) structure after optimization. The vacancy is partially filled upon the rotation of the molecules. The positions of the dislocation lines are marked by S

This model was constructed with a supercell of 8 × 1 × 8 unit cells and the removal of four neighbouring molecules parallel to [101] [Fig. 11 ▸(*a*)]. During the optimization, the molecules show a clear tendency to fill the vacancy by rotations of the entire molecules. The rotations start with the molecules in the layers close to the dislocation line, and continues with the layers in between. Fig. 11 ▸(*b*) shows the situation without reaching full convergence. The energy of this structure is by 243.8 kJ mol^−1^ per dislocation line higher than the energy of the undisturbed quinacridone; hence this structure is energetically very unfavourable.

#### 5.2.4. Dislocation line parallel to [010] and Burgers vector (0,0,1)

**Figure 12:**
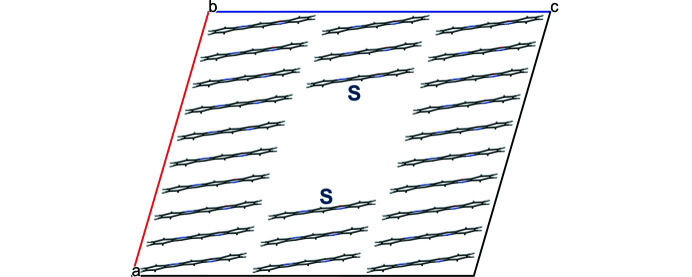
Edge dislocation along [010], Burgers vector (0,0,1).

The dislocation loop was constructed in a supercell of 10 × 1 × 3 unit cells by the removal of four neighbouring molecules in the [100] direction (see Fig. 12 ▸). The molecules barely move into the vacancy, making this model energetically unfavourable with an energy of 115.07 kJ mol^−1^ per dislocation line.

#### 5.2.5. Dislocation line parallel to [001] and Burgers vector (1,0,0)

For this case, several models were constructed, which differ in the relative position of the two dislocation lines and the number of withdrawn molecules.

**Figure 13:**
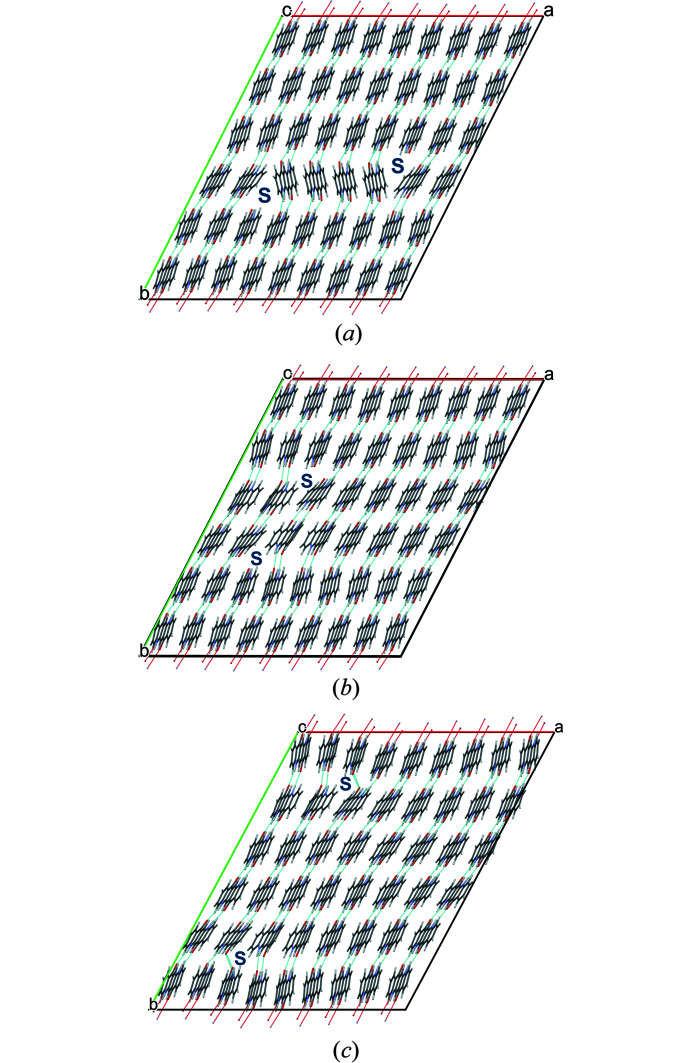
Models for an edge dislocation along [001] with a Burgers vector (1,0,0): (*a*) Model 1, (*b*) Model 2 and (*c*) Model 3. The positions of the dislocation lines are marked by S.

In **Model 1**, the dislocation lines are separated by 4 ***a***_0_ − ***b***_0_, and one molecule was removed [see Fig. 13 ▸(*a*)]. In the optimized structure the hydrogen bonds are disrupted at the dislocation line, otherwise they are maintained through rotation and translation of the molecules. Although this structure looks chemically reasonable, the energy increase is high (210.8 kJ mol^−1^). Apparently, the distortion of the structure causes a significant increase of the intermolecular energy. The removal of a second molecule increases the energy further by 245.5 kJ mol^−1^.

In **Model 2**, the dislocation lines are separated by 2 ***b***_0_, and two molecules were removed. In the optimization, this void is filled by the neighbouring molecules [Fig. 13 ▸(*b*)], which is in contrast to the maintenance of the structure observed with a single vacancy [Fig. 4 ▸(*c*)]. Some of the hydrogen bonds become distorted. The energy increase is 156.9 kJ mol^−1^ per dislocation line and per missing molecule, which is slightly less than for a single vacancy (233.4 kJ mol^−1^).

**Model 3** exhibits a similar behaviour. Here, the dislocation lines are separated by 4 ***b***_0_ with the removal of four molecules. The optimized structure [Fig. 13 ▸(*c*)] is a good impression of the actual local structure at an edge dislocation. The energy is 161.3 kJ mol^−1^ per dislocation line, comparable to Model 2.

#### 5.2.6. Dislocation line parallel to [001], Burgers vector (0,1,0)

Akin to a dislocation line parallel to [100] with a Burgers vector (0,1,0) (see Section 5.2.1), this lattice defect leads to a disruption of many hydrogen bonds and is energetically very unfavourable.

### 5.3. Screw dislocations

In screw dislocations, the Burgers vector is parallel to the dislocation line.

#### 5.3.1. Dislocation line along [100]

**Figure 14:**
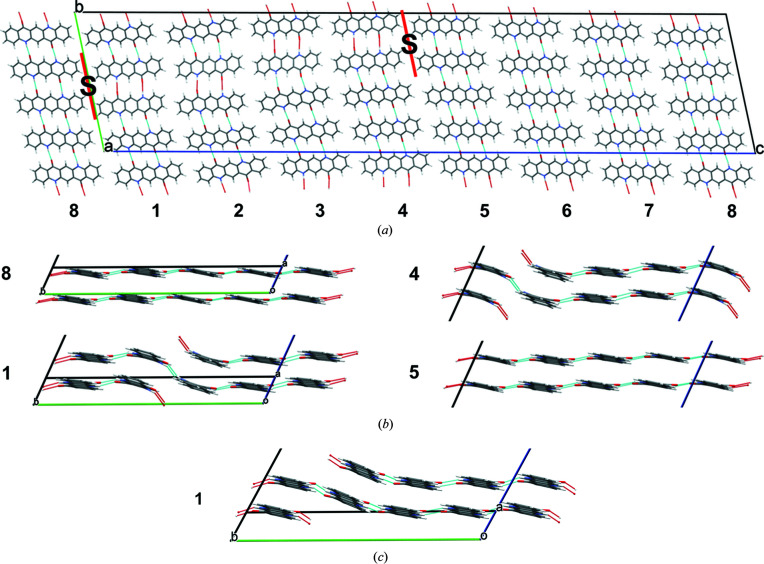
Screw dislocation with the dislocation line along [100] with supercell 1 × 4 × 8. (*a*) View direction [100]. The positions of the dislocation lines are marked by 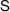 . The slip planes are drawn in red. The numbers 1–8 denote the chain numbers. (*b*) Local structure at the chains 8/1 and 4/5. View direction 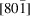 (which corresponds to 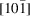 in the original cell). (*c*) Starting structure of chain 1.

The investigated structure is based on a 1 × 4 × 8 supercell with two dislocation lines with the Burgers vectors (1,0,0) and 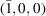 . The slip plane is (001), see Fig. 14 ▸. The starting model was constructed with chemically reliable chains having reliable hydrogen bond geometries [see Fig. 14 ▸(*c*) for chain No. 1]. During the optimization, the molecules shifted along [100], resulting in a structure with strongly distorted hydrogen bonds [see Fig. 14 ▸(*b*)]. Due to the distortion, the energy is 61.6 kJ mol^−1^ per dislocation line, although no hydrogen bonds are broken.

#### 5.3.2. Dislocation line along [010]

**Figure 15:**
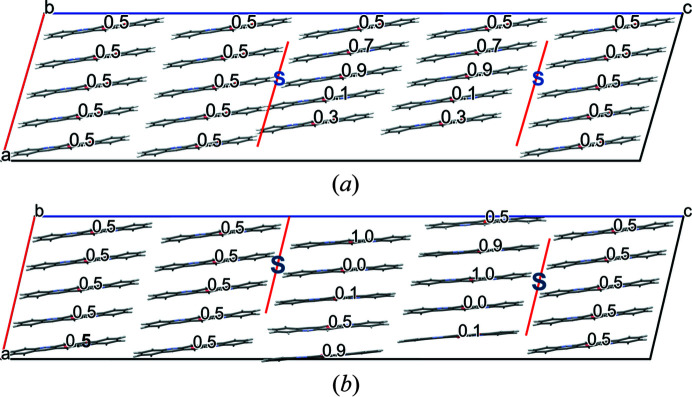
Screw dislocation with the dislocation line along [010]. The numbers denote the *y*-coordinate of the molecular centres. The positions of the dislocation lines are marked by S. The slip planes are drawn in red. View direction [010]. (*a*) Starting structure, (*b*) optimized structure.

A screw dislocation along [010] does not break any hydrogen bonds, but entire chains of molecules are shifted in the chain direction. Our model has a slip plane parallel to (001) (see Fig. 15 ▸). The starting model consisted of an ensemble with various shifts in the *y*-direction (0.1, 0.3, 0.5, 0.7, 0.9) [see Fig. 15 ▸(*a*)]. In the optimized structures, all molecules moved to positions close to *y* = 0 or 0.5 [see Fig. 15 ▸(*b*)]. Apparently, these positions are energetically preferred.

Apart from the longitudinal translation of the chains, the structure is barely distorted. Accordingly, the lattice energy is quite favourable with Δ*E* = 38.0 kJ mol^−1^, because the hydrogen bonds are not broken and the van der Waals energy as well as the dense packing with π–π interactions are maintained.

#### 5.3.3. Dislocation line along [001]

The [001]-direction corresponds to the long molecular axis. A screw dislocation along [001] causes a translation of the molecules along their long axis leading to the loss of hydrogen bonds, and unfavourable intermolecular contacts. Hence screw dislocations in this direction would be energetically very unfavourable.

### 5.4. Misorientation of an entire chain

#### 5.4.1. Chain direction [010]

**Figure 16:**
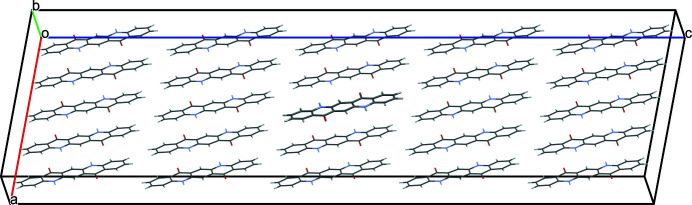
Supercell with misoriented chain after optimization. View along b*. The misoriented chain is drawn in bold.

In [010] direction, the molecules are connected by hydrogen bonds into a chain. The rotation of all molecules in the chain by 180° around their medium molecular axis, which is almost parallel to [010], corresponds to a rotation of an entire chain around [010] by 180° (see Fig. 16 ▸). This rotation leads to a mutual exchange of CO and NH groups of all molecules in the chain. The same structure is formed by mirroring of the chain at a mirror plane perpendicular to [010]. These operations do not break any hydrogen bonds. Furthermore, the terminal benzene rings of the molecule do not move, *i.e.* the outer shape of the chain does not change. The chain itself has rod group 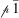 (rod group No. 2; Kopský & Litvin, 2002 ▸). In contrast, the outer shape of the chain is close to having 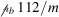 symmetry, which is a non-standard setting of 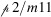 (rod group No. 6).

Correspondingly, the van der Waals interactions between the benzene rings at the end of the molecules is in the **c** direction do not change. However, the interaction with neighbouring molecules in the **a** direction (π stacking) is modified because the van der Waals interactions as well as the electrostatic interactions change. Surprisingly, this reorientation leads to an energy increase of only 1.57 kJ mol^−1^. Apart from the misorientation of the chains, the crystal structure is not distorted at all. The low energy indicates that this lattice defect should frequently occur in the real crystal. Correspondingly, the crystals may contain different arrangements of the same rod. If these arrangements were periodic, they would be rod polytypes. However, the distribution of misoriented chains is not periodical, but almost statistical.

#### 5.4.2. Other directions

The misorientation of a row of molecules in any other direction leads to unfavourable C=O⋯O=C and N—H⋯H—N contacts, which are energetically very unfavourable as shown in Section 4.4.

### 5.5. Misorientation of two neighbouring chains

As seen in Section 5.4.1, the misorientation of an entire chain in the [010]-direction causes an energy increase of only 1.57 kJ mol^−1^. This prompted us to investigate if an ensemble of two neighbouring misoriented chains might be even more favourable. Each chain is surrounded by six neighbouring chains with mutual translation vectors of (1,0,0), (0,0,1), 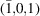 , 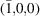 , 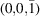 and 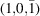 . Correspondingly, there are three possibilities to group two neighbouring chains, which are described below.

#### 5.5.1. Neighbours with translation of (1,0,0)

**Figure 17:**
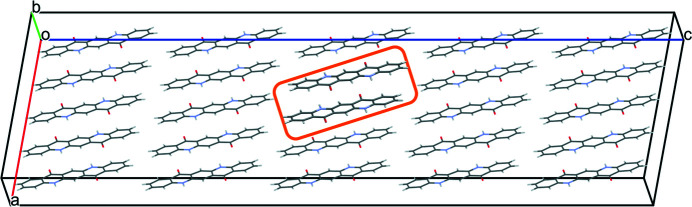
Two misoriented chains associated by a translation of (1,0,0). The orange box highlights the misoriented chains. View along ***b*** *.

The simultaneous rotation of two neighbouring chains associated by a translation of (1,0,0) leads to an energy of 1.33 kJ mol^−1^ per chain, which is even slightly more favourable than the rotation of two separate chains (Fig. 17 ▸).

#### 5.5.2. Neighbours with translation of (0,0,1)

**Figure 18:**
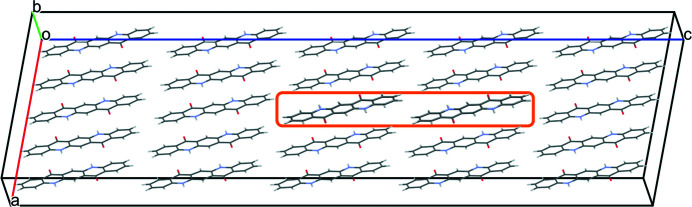
Two misoriented chains associated by a translation of (0,0,1). The misoriented chains are framed in orange. View along b*.

Two chains with a mutual shift of (0,0,1) are connected only by a single van der Waals contact of two CH groups (see Fig. 18 ▸). Correspondingly, the energy (1.59 kJ mol^−1^ per chain) is about the same as for separate chains.

#### 5.5.3. Neighbours with translation of (1,0,1)

**Figure 19:**
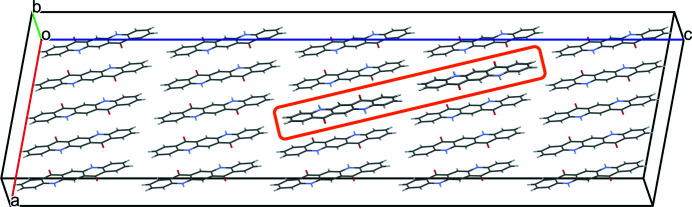
Two misoriented chains associated by a translation of 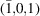 . The misoriented molecules are framed in orange. View along ***b*** *.

As in the previous model, the two chains are connected only by a single van der Waals contact of two CH groups (see Fig. 19 ▸). Surprisingly, this arrangement of two misoriented chains is even better than that of two chains linked by π–π stacking (Section 5.5.1). The energy is 1.28 kJ mol^−1^.

Apparently, the interactions between the terminal CH groups play a non-negligible role. The low energies of all sets of neighbouring chains indicate that a real crystal may also contain larger sets of misoriented chains, *e.g.* misoriented layers or volume regions, which will be described in Sections 6.2 and 7.2.

## 6. Planar defects

A simple chain structure can, in principle, exhibit various types of planar defects. We will focus on a few interesting ones.

### 6.1. Small-angle grain boundaries

A small-angle grain boundary interrupts the 3D translational periodicity of the crystal. For a calculation of a small-angle grain boundary with a 3D periodic model, the model must contain at least two small-angle grain boundaries, so that the sum of the Burgers vectors vanishes. A small-angle grain boundary can be regarded as a series of parallel edge dislocations. Actually, a periodic series of parallel edge dislocations have already been investigated in Section 5.2. The resulting energies were very high, which is an argument against the occurrence of small-angle grain boundaries.

### 6.2. Misorientation of a layer of molecules

As seen in Section 5.4.1, the rotation of an entire chain of molecules around [010] by 180° leads to an energy increase of only 1.57 kJ mol^−1^. Similarly, all molecules of an entire layer can be rotated around [010] by 180°, leading to a layer with inverted molecular orientation. The resulting model can be regarded as a special case of a stacking fault, in which the molecules are rotated, but the molecular centres do not move. Hence the translational periodicity of the molecular centres is maintained.

It should be noted, that the rotation of molecules by 180° has a different effect than a twinning by reflection at a net plane, because the structure is triclinic, and a mirroring at a net plane would change the molecular orientations and positions in a different way, see Sections 6.3 and 8.

In this section, we describe the misorientation of molecules in a single layer. Models with a misorientation of a block of molecules in neighbouring layers are described in Section 7.2.

#### 6.2.1. (100) layer

**Figure 20:**
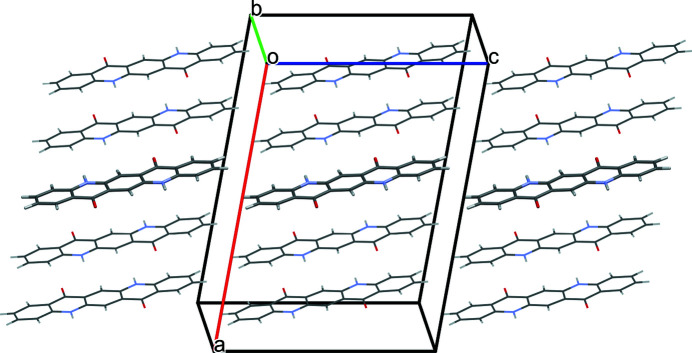
Layer of misoriented molecules, parallel to (100). View along ***b*** *. The misoriented layer is shown in bold.

An inverted orientation of molecules in a layer parallel to (100) (see Fig. 20 ▸) leads to an energy increase of only 1.46 kJ mol^−1^ per molecule, which is similar to the instance with a single misoriented chain.

#### 6.2.2. (101) layer

**Figure 21:**
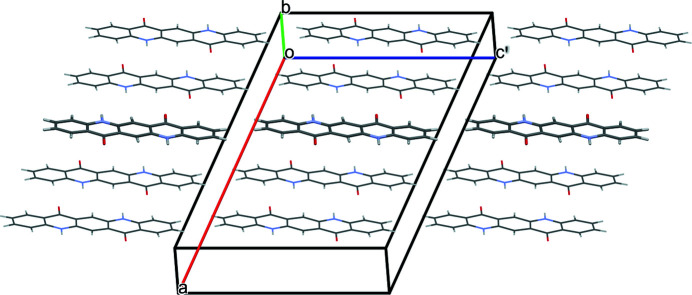
Layer parallel to (101) with inverted orientation, calculated as a (100) layer in a unit cell with 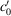 = *c*_0_ − *a*_0_. View along ***b*** *.

As seen in Section 5.5, the chains are not only linked in the [001] direction, but also in the 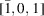 direction, which corresponds to a layer parallel to (101). The model for a structure with a (101) layer with inverted molecular orientation was constructed in the following way: the unit cell was transformed with 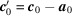 . The (101) layer of the original cell corresponds to the (100) layer of the transformed cell. Subsequently, a 5 × 1 × 1 supercell was assembled and one molecule was rotated (see Fig. 21 ▸). After optimization with free unit-cell parameters, the energy was only 0.78 kJ mol^−1^ per molecule higher than that of an undisturbed crystal. The unit-cell parameters only changed by less than 0.17 Å and 1.1°.

#### 6.2.3. (001) layer

**Figure 22:**
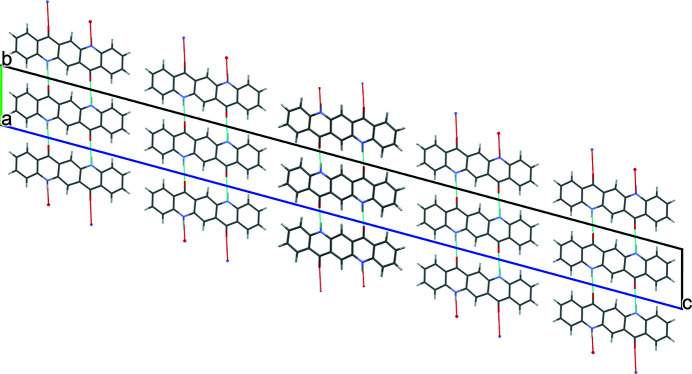
Layer parallel to (001) with inverted molecular orientation. View direction [100]. The central layer consists of molecules with inverted orientation.

The rotation of molecules within a layer parallel to (001) (see Fig. 22 ▸) leads to an energy increase of only 1.34 kJ mol^−1^ per molecule, similar to a rotation in a (100) layer.

### 6.3. Other stacking faults

#### 6.3.1. Herringbone instead of parallel stacking

**Figure 23:**
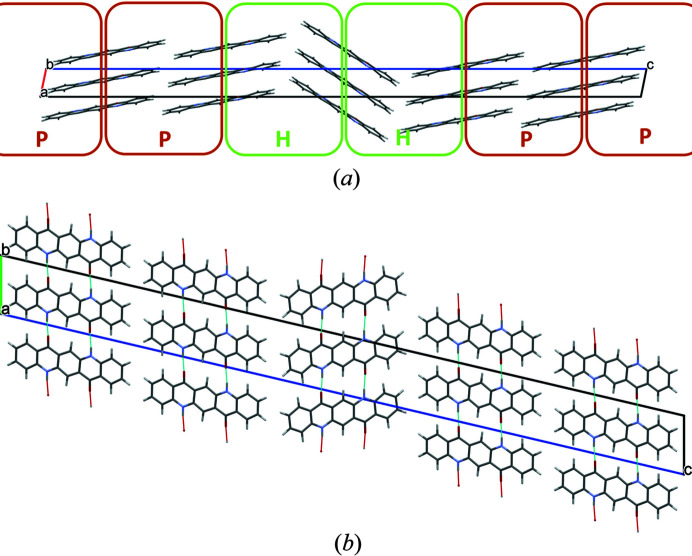
Structure of a stacking fault with local herringbone stacking. (*a*) View direction [010]. Herringbone contacts are marked with an H, parallel contacts with a P. (*b*) View direction [100].

A mixture of herringbone and parallel stacking of layers along (001) was experimentally observed in α^II^-quinacridone by electron diffraction. The α^II^-phase exhibits predominantly herringbone stacking with a minor contribution of parallel stacking [see Fig. 3 ▸(*a*)]. In contrast, the α^I^-phase shows mainly parallel stacking. However, stacking faults with herringbone stacking cannot be ruled out. A corresponding model is shown in Fig. 23 ▸. The herringbone fragment corresponds to the structure of the α^II^-phase, except for a misorientation of the central chain.

The herringbone fragment in the α^II^-phase corresponds to a twinning at (001) with a twin domain thickness of one layer. Twins at (001) with a larger domain thickness are described in Section 8.1.

The stacking fault in α^I^-quinacridone leads to an energy increase of 4.36 kJ mol^−1^, corresponding to 2.18 kJ mol^−1^ per herringbone contact, which is slightly higher than for a pure herringbone stacking of the α^II^-phase (Δ*E* = 1.53 kJ mol^−1^).

#### 6.3.2. Commensurate misfit-layer structures, *i.e.* stackings with different lateral periodicities

**Figure 24:**
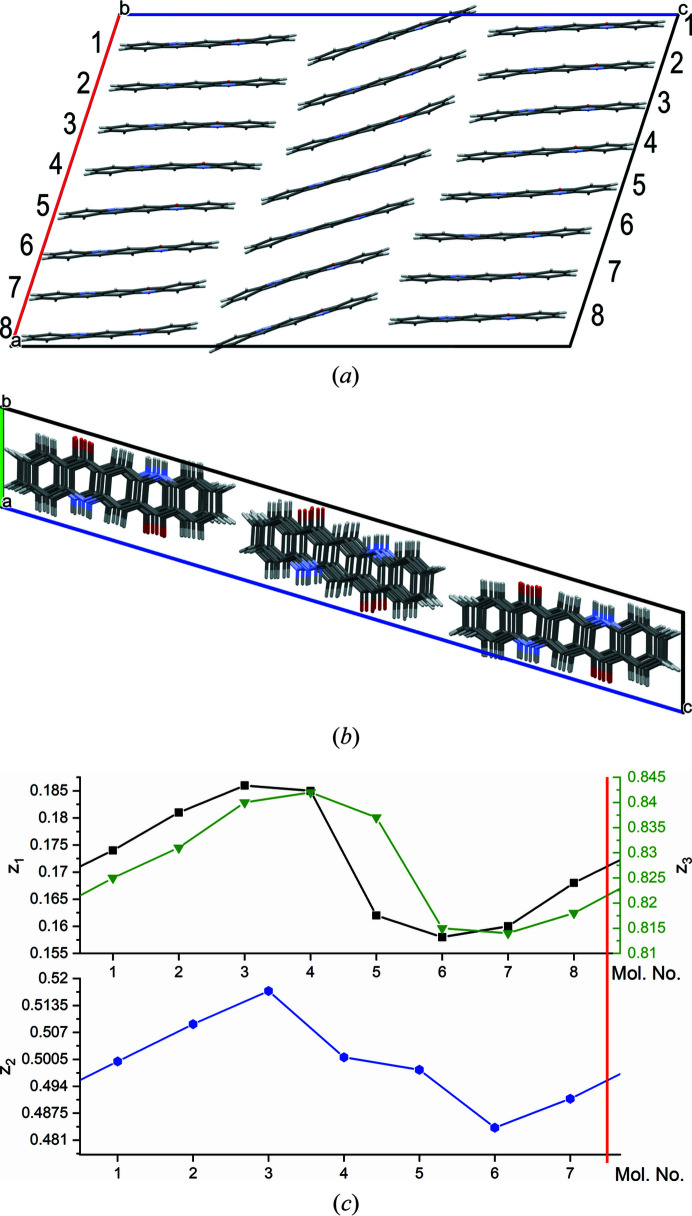
Views of misfit-layer structure: (*a*) in direction [010] and (*b*) in direction [100]. (*c*) Modulation of the molecular position in the *z* direction. Top curves: molecules in the 8-layer, bottom: molecules in the 7-layer.

Fig. 24 ▸ shows a three-layer model in which the first and third layers contain eight molecules and the second one only seven. This model was obtained by chance. In our investigation of structures with a missing chain, we constructed a 8 × 1 × 3 supercell and removed the central molecule, which corresponds to the removal of an entire chain along [010]. In the subsequent optimization with free unit-cell parameters, the molecules of the central layer rotated by 13.73°, and the unit-cell parameter ***c*** shrank by 0.21 Å. Despite of the missing molecule, the density is almost as high as in the undisturbed structure (1472 versus 1493 kg m^−3^). The resulting structure is shown in Fig. 24 ▸. This structure contains eight molecules per supercell in the first and third layer but only seven molecules in the central layer, which corresponds to a commensurate misfit-layer structure with 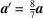 . In inorganic chemistry, such structures are usually modulated. In the case of molecular crystals, the modulation could affect the molecular position, orientation, and conformation (Wagner & Schönleber, 2009 ▸). Our model is modulated, too, especially concerning the shift of the individual molecules in the **c** direction, which is clearly visible in Fig. 24 ▸(*a*). The modulation curve is shown in Fig. 24 ▸(*c*). The energy of this structure is 76.7 kJ mol^−1^ for the supercell with 8+7+8 molecules, corresponding to 11.0 kJ mol^−1^ per molecule in the central layer.

For inorganic compounds, such structures containing layers with atom deficiencies are known, for example the Magnéli phase Mo_8_O_23_ (Magnéli, 1948 ▸). Misfit-layer structures are known in inorganic chemistry, *e.g.* structures built from two chemically different layers with different lateral dimensions (Makovicky & Hyde, 1981 ▸). Similarly, organic chain-misfit structures are known for host–guest systems. In contrast, to the best of our knowledge, organic misfit-layer structures have not been observed experimentally for homomolecular organic compounds. There are several problems with such structures:

(1) Mixed oxidations states, such as Mo^VI^/Mo^V^ in Mo_8_O_23_, are rare in organic compounds.

(2) The individual planes may very easily slip on each other. Consequently, the crystal is probably mechanically unstable.

(3) At the surface of a growing crystal, such a slip may occur very easily, and an additional molecule can be inserted in the deficient layer to achieve the energetically more favourable periodic stacking. This process is even facilitated by the typical high mobility of molecules on growing surfaces.

(4) According to the energy, such misfit layers are very rare. In the diffraction patterns, such a low number of misfit layers would cause only very minor effects (faint diffuse scattering), which would be too weak to be observed experimentally. Also in the pair-distribution function, the effect is too small to be recognised.

In spectroscopic methods (IR, Raman, SS-NMR, UV–vis *etc.*) the low concentration of misfit layers is probably below the detection limit. Furthermore, the layer misfit does not change the hydrogen bond system and causes only a small shift in the spectra. Only HRTEM, AFM or similar local methods would work.

## 7. Volume defects

### 7.1. Voids (three-dimensional vacancy aggregates)

Three-dimensional vacancy aggregates are energetically unfavourable, as already established for aggregates of line vacancies (see Section 5). In a real crystal, such voids will typically be filled with water or other molecules.

### 7.2. Domains of misoriented molecules

The crystal may contain entire regions with inverted orientation of. Two situations were considered: (i) large sheets of misoriented molecules with the sheets being parallel to (001), (ii) blocks of 4 × 4 misoriented chains parallel to [010].

#### 7.2.1. Lamellar domains with misoriented molecules

**Figure 25:**
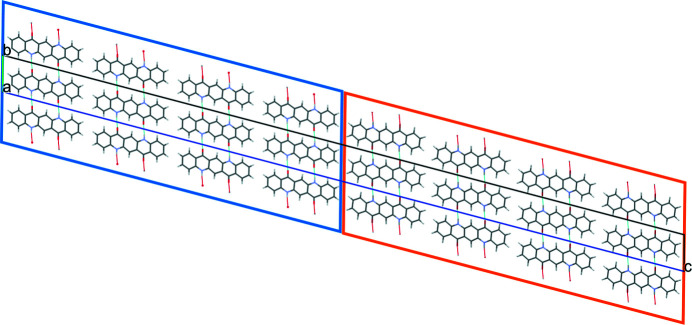
Lamellar domains with rotated molecules. Blue and orange boxes mark the domains of the different molecular orientations. View direction [100].

Fig. 25 ▸ shows a crystal assembled from a periodic arrangement of domains, in which each domain consists of a sheet with a thickness of four molecules with inverted orientation of the molecules. Note that this model does not reflect a twinning, but only a rotation of the molecules. A twin would change the direction of the lattice vectors, but in our model, the vectors of the triclinic lattice were not modified. In the subsequent optimization, the inverted orientation did not cause a change in the molecular positions, and the general packing did not change, even the unit-cell parameters changed by less than 0.2 Å (see Table S4). The energy of the supercell increases by 4.50 kJ mol^−1^, *i.e.* by 2.25 kJ mol^−1^ per phase boundary.

#### 7.2.2. Blocks of misoriented molecules

**Figure 26:**
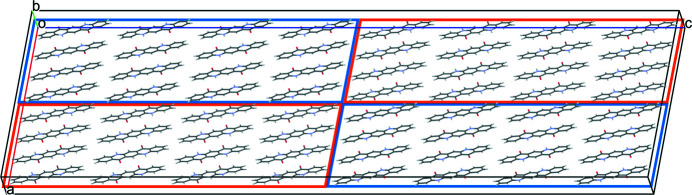
Blocks of misoriented molecules. The domains with a different orientation are marked with orange and blue. View direction ***b*** *.

Fig. 26 ▸ shows a model which contains of blocks of 4 × 4 inverted chains parallel to [010]. As for all other models with inverted chains, neither the position nor the spatial orientation changes significantly. The resulting structure has an energy of 36.0 kJ mol^−1^ per supercell. The supercell contains 32 modified molecule–molecule contacts at the domain boundaries; hence, the energy increase is as low as 1.1 kJ mol^−1^ per modified molecule–molecule contact.

## 8. Twinning

A simple triclinic compound such as α^I^-quinacridone can exhibit twinning in various ways. Here we focus on those twins which might explain the twinning observed in the HRTEM image (see Section 10).

### 8.1. Twinning by mirroring at (001), Model 1

**Figure 27:**
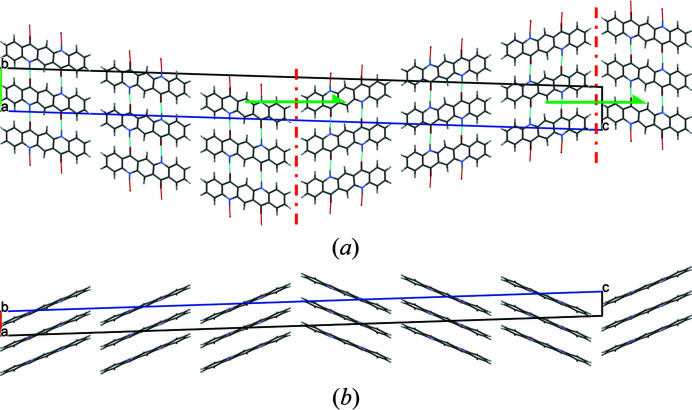
Twinning by mirroring at (001), Model 1. (*a*) View direction [100]. The local glide planes between the twin domains are drawn in red, the local screw axes in green. (*b*) View direction [010].

Twinning of the crystal structure of α^I^-quinacridone at the (001) face corresponds to a mirroring of the structure at a plane parallel to (001), whereby the mirror plane is located between the molecules. The lattice-energy optimization reveals that a mirror plane would actually lead to unfavourable C—H⋯H—C contacts. An energetically more favourable local structure is formed by replacing the mirror plane with a local glide plane parallel to (001), having an intrinsic translation of about (−0.42, 0.24, 0) (see Fig. 27) ▸.

Since the individual twin domains have inversion symmetry, there is a second possibility to describe this local structure at the twin boundary: The combination of the local glide plane with the inversion centre generates a local screw axis parallel to ***c*** * with an intrinsic translation of 1/***c***_0_* [Fig. 27 ▸(*a*)].

In the twin structure, the molecular planes in the different twin domains are inclined to each other by 44.9°. If the second twin domain has a thickness of one molecule only, the structure corresponds to the herringbone stacking fault described in Section 6.3.1, except for a misorientation of the central chain. If nearly all domains consist of only one layer of molecules, the structure of the α^II^-phase is obtained.

The twin was modelled using domains with a thickness of three molecules – which should be sufficient. Optimization leads to an energy as low as 0.92 kJ mol^−1^ per twin domain. Hence, this microscopic twinning is energetically very favourable.

### 8.2. Twinning by mirroring at (001), Model 2

**Figure 28:**
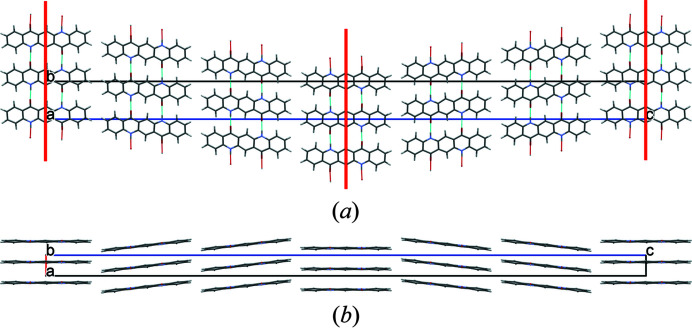
Twinning by mirroring at (001), Model 2. (*a*) View direction [100]. The local mirror planes are drawn in red. Note that the mirror planes do not act on the molecules, which they cut, but only on the neighbouring and next-neighbouring molecules. (*b*) View direction [010].

In the previous section, the twinning mirror plane parallel to (001) was located between the molecules. Alternatively, the mirror plane can go through the molecular centres. Since the molecules themselves do not possess a corresponding mirror plane, they must be disordered. However, this disorder leads only to a mutual exchange of CO and NH groups within an entire layer of molecules, which has no great effect on the packing energy, as shown in Section 6.2.3. The adjacent layers follow an exact mirror image, see Fig. 28 ▸.

After optimization, the molecules form a wavy arrangement [see Fig. 28 ▸(*b*)]. This structure has an energy of 6.4 kJ mol^−1^ per twin boundary.

### 8.3. Twinning by mirroring at (001), Model 3

**Figure 29:**
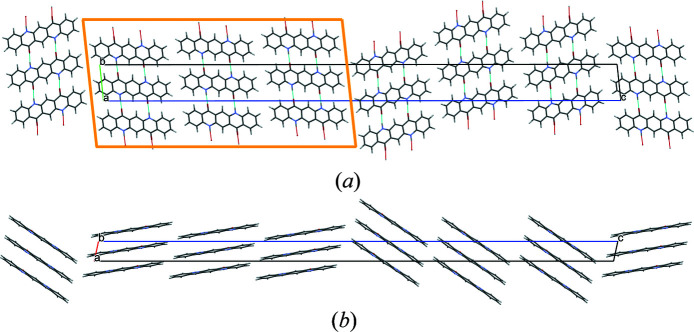
Attempt to construct a structure with twinning by mirroring at (001), Model 3. (*a*) View direction [100]. (*b*) View direction [010]. The left domain, marked by an yellowish orange box, has a different structure than α^I^-quinacridone.

A third attempt to construct a twin by mirroring the structure at the (001) face leads to the structure shown in Fig. 29 ▸. This model has an energy of only 0.77 kJ mol^−1^ per twin boundary. On a first glance, the structure seems to be reasonable. However, the two twin domains have a different structure. The ‘left’ twin domain does not correspond to the crystal structure of α^I^-quinacridone, but to a different, hypothetical polymorph, which has not been observed experimentally.

## 9. Discussion: probability of lattice defects

If crystals are grown under thermodynamic control, the frequency of the individual lattice defects can be roughly estimated with the Boltzmann formula:

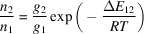

where 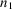 , 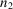 are probabilities of two local structures, *g* is degeneracy and 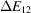 is energy difference between the two local structures. All investigated point defects (vacancy, interstitial molecule, misoriented molecule) are energetically so unfavourable that they will hardly occur. Point defects with foreign molecules (*e.g.* incorporation of water or byproducts) may occur, but were not investigated here.

Similarly, all investigated edge dislocations and screw dislocations are energetically very unfavourable. The only exception is a screw dislocation along [010], which is more favourable because it keeps the hydrogen bonded chains intact and consists only of a translation of the chains along the chain direction. This defect has a relative energy of 38.0 kJ mol^−1^ per dislocation line, corresponding to a frequency of 5.3 × 10^−8^, which indicates that this lattice defect might very rarely occur in a real crystal.

In contrast, the rotation of an entire chain of molecules along the chain axis [010] leads to a surprisingly low energy increase of only 1.57 kJ mol^−1^ per chain molecule. With the Boltzmann formula one may estimate that about one third of the chains have an inverted orientation. Calculations with two neighbouring chains reveal that this probability even slightly increases if two neighbouring chains are rotated simultaneously.

Similarly, the rotation of all molecules within an entire layer, or within a sheet consisting of several layers or within a block of chains parallel to [010], only requires a small energetic effort. Correspondingly, such defects can easily occur. The rotation of all molecules within a layer corresponds to a stacking fault in which the molecules are rotated, but the 3D-translational periodicity of the molecular positions is left unchanged.

Overall, the crystal should contain approximately one third of chains with inverted orientation. These misoriented chains are distributed almost statistically, with only a weak correlation between neighbouring chains. However, all these orientation defects do neither significantly change the unit-cell parameters, nor the molecular positions. This disorder does not lead to a periodic superstructure – even not to a local superstructure – and correspondingly, not to any superstructure reflections, but only to some diffuse scattering. Since the disorder affects only the exchange of CO by NH groups, the diffuse scattering should be quite weak. In single crystal data, the orientational disorder would surely be apparent from the electron density distribution at the NH and CO groups. In contrast, in the powder diffraction pattern of this nanocrystalline material, the disorder is not visible.

There are two additional lattice defects which are energetically accessible: herringbone stacking faults and twinning:

(1) The parallel stacking can be interrupted by a layer with herringbone stacking. Such a mixture of parallel and herringbone stacking of chains was already observed in α^II^-quinacridone. There, the herringbone stacking dominates, and is interrupted by layers with parallel stacking. The energy differences between parallel and herringbone stacking are low, in the α^I^-phase as well as in the α^II^-phase. This suggests a continuous series of structures from a pure parallel packing to a pure herringbone packing, with the α^I^ and α^II^ phases being close to both ends of this series.

(2) The same local structure is observed in twinning along (001). The only difference is that in a twinning the domains are larger than in a single layer. This twinning requires an energy increase of 0.92 kJ mol^−1^ only, hence it should frequently occur in a real crystal.

## 10. Experimental observation of lattice defects by HRTEM

**Figure 30:**
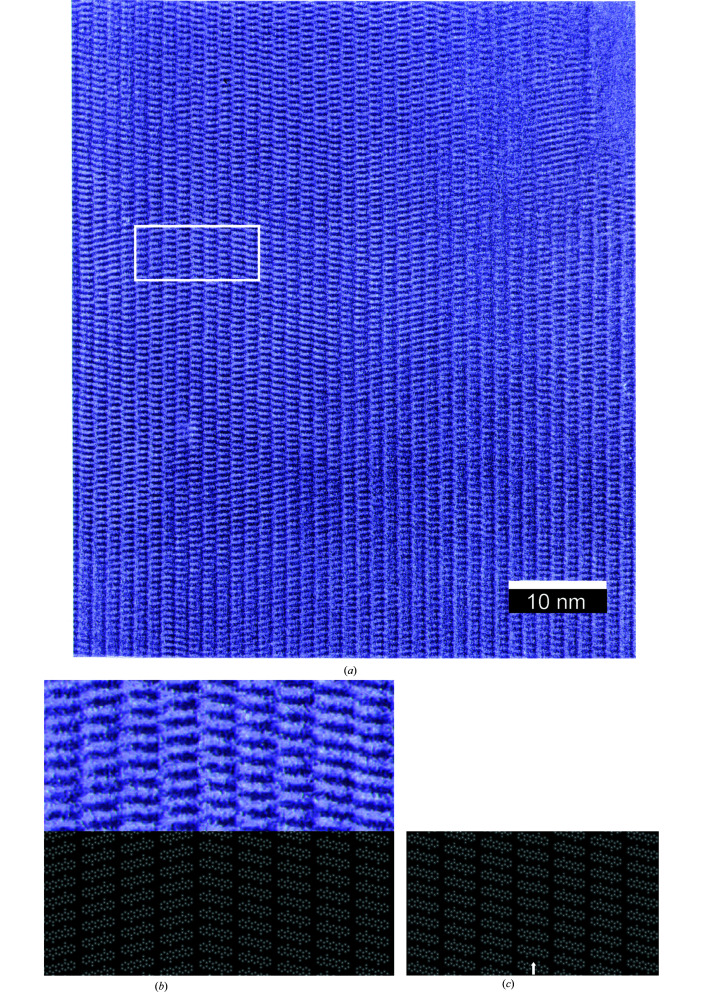
(*a*) HRTEM image of α^I^-quinacridone, with kind permission of T. Ogawa. View direction [100], ***b*** axis vertical. The white box marks the section enlarged in (*b*). (*b*) Section of the HRTEM image, and the corresponding simulated twin Model 1. (*c*) Simulated structure containing a layer (100) with misoriented molecules. The layer is marked by an arrow.

A highly crystalline sample of α^I^-quinacridone was obtained by Tetsuya Ogawa *et al.* by vacuum deposition of purified quinacridone on alkali halide single crystals at 140–170°C (Ogawa *et al.*, 1999 ▸). Single crystals of sizes up to 700 × 100 × 20 nm were grown. The HRTEM image of one of the crystals was kindly provided by Tetsuya Ogawa. The image is shown in Fig. 30 ▸.

At first glance, the molecules seem to be well ordered in this HRTEM image. However, the HRTEM image actually shows many lattice defects. For example, the region marked by a white box in Fig. 30 ▸(*a*) contains a twin boundary, which resembles the simulated twin Model 1 [see Fig. 30 ▸(*b*)].

The resolution of the image does not allow the observation of finer defects. For example, a misorientation of a chain of molecules has an effect too weak to be seen in the HRTEM. Even an entire layer of misoriented molecules could hardly be recognized [see Fig. 30 ▸(*c*)]. Correspondingly, the HRTEM image does not provide information on the frequency of misoriented chains. Point defects such as vacancies or small foreign molecules are too weak to be seen. Interstitial molecules would be recognizable through the distortion of the structure in their vicinity, but are absent in the HRTEM image. (A similar effect would be produced by radiation damage to the crystalline structure during TEM imaging, and, therefore would be difficult to interpret.) Other larger defects, such as edge or screw dislocations, grain boundaries, or aggregates of foreign molecules are either absent or not visible.

## 11. Lattice defects in industrial α^I^-quinacridone

All the simulations in this paper imply a crystal growth near the thermodynamic equilibrium. The single crystals obtained by Ogawa *et al.* (1999 ▸) were grown by vacuum sublimation. Nevertheless, they contain a high number of lattice defects. In the industrial process, the crystallization of α^I^-quinacridone is very far from thermodynamic equilibrium: a hot solution of probably protonated quinacridone in molten polyphospho­ric acid at 150°C is poured into ice water, whereby the quinacridone instantaneously precipitates as an insoluble, nanocrystalline, crude powder. Under these conditions, the crystals surely contain a high number of defects, including those which were calculated to be energetically unfavourable, *e.g.* various types of point defects, edge and screw dislocations. Even fragments of misfit-layer structures cannot be ruled out.

## 12. PDF investigations on α^I^-quinacridone

We tried to elucidate the nature of lattice defects in industrial α^I^-quinacridone using pair-distribution function (PDF) analysis (Schmidt, 2010 ▸).

**Figure 31:**
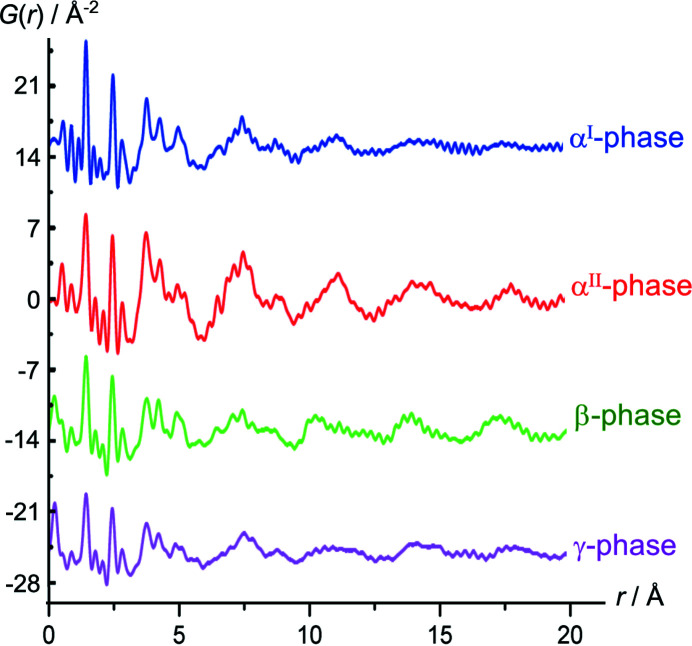
Pair-distribution functions of quinacridone polymorphs (Schmidt, 2010 ▸).

The PDFs of the four quinacridone polymorphs are significantly different (see Fig. 31 ▸). However, the α^I^-phase contains so many molecules at surfaces, grain boundaries, probably also in amorphous regions and at various lattice defects that it is impossible to disentangle all these effects. Furthermore, a misorientation of an entire chain, which is calculated to be very frequent, has only a very minor effect to the PDF, because most atoms remain on their original positions.

Hence, the PDF does not provide information on the individual lattice defects.

## 13. Conclusion

**Figure 32:**
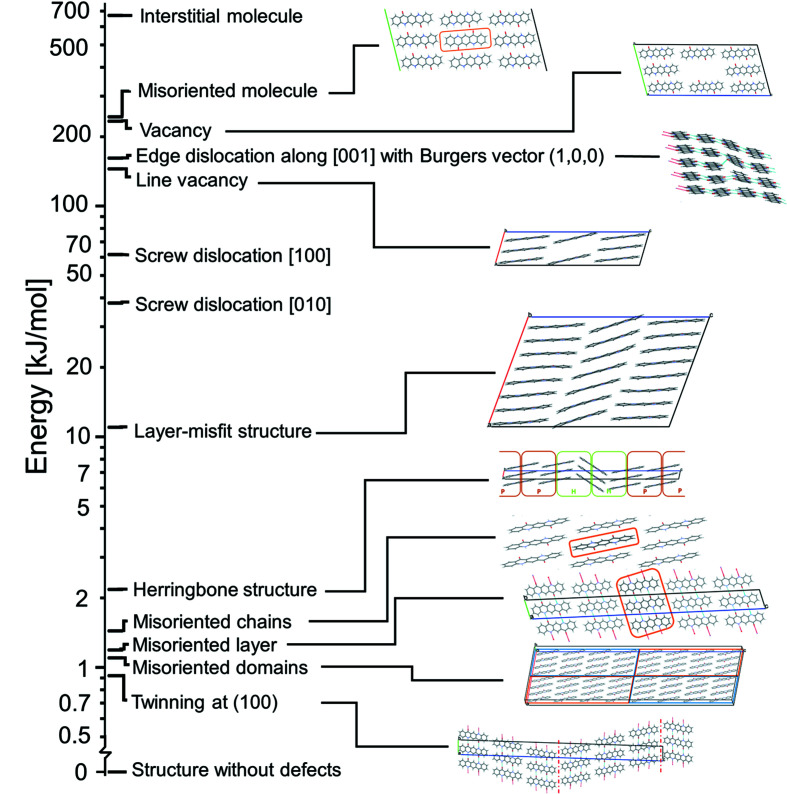
Overview of the major lattice defects described in this paper, sorted according to energy.

In this work, a large variety of lattice defects in α^I^-quinacridone was investigated, showing energies and local structures in the vicinity of the defects. An overview is given in Fig. 32 ▸. Surprisingly, a rotational flip of an entire chain of molecules is a very facile lattice defect, having an energy of about 1.5 kJ mol^−1^ only. Correspondingly, about one third of the chains is expected to be misoriented in a real crystal. The ease of the misorientation is caused by the symmetry of the molecular shape, which is close to having an additional mirror plane. Furthermore, twinning at the (001) plane requires only 0.92 kJ mol^−1^. According to the HRTEM image, this twinning occurs frequently. Other lattice defects, such as vacancies, interstitial molecules, edge and screw dislocations, are energetically quite unfavourable. Nevertheless, they may occur in a real crystal, if the crystallization occurs far from thermodynamic equilibrium.

We expect that these results can be transferred to other organic structures with chains of molecules. It should be noted that the misorientation of a molecular chain is only possible if the chain has a nearly pseudosymmetric shape. All other lattice defects can be expected to be as rare as in α^I^-quinacridone.

## Supplementary Material

Crystal structure: contains datablock(s) I, commensurate-structure, Twin-Model_1. DOI: 10.1107/S205252062200779X/ra5112sup1.cif

Supporting information file. DOI: 10.1107/S205252062200779X/ra5112sup1.pdf

CCDC references: 2194358, 2194359, 2194360

## Figures and Tables

**Table 1:** Evaluation of the force field Lattice-energy optimizations on α^I^-quinacridone without lattice defects. ‘Dreiding’ denotes the Dreiding force field with 6-31G**-ESP charges and Ewald summation.

	Experimental†	Dreiding	DFT-D
*a* (Å)	3.8017 (15)	3.9241	3.685
*b* (Å)	6.901 (3)	6.8938	6.386
*c* (Å)	14.766 (6)	14.9598	14.574
α (°)	99.30 (8)	98.999	101.486
β (°)	99.74 (6)	100.601	97.861
γ (°)	110.48 (6)	114.976	100.066
*V* (Å^3^)	346.67 (11)	347.917	325.646

† After unit-cell transformation.
