# Supplementary material for: Lattice defects in quinacridone
Source: Acta Crystallogr B Struct Sci Cryst Eng Mater. 2022 Sep 9;78(Pt 5):763–80. doi: 10.1107/S205252062200779X (PMC9533312; doi:10.1107/S205252062200779X)
Supplement: Supplementary file 2 [file b-78-00763-sup1.pdf]

STRUCTURAL SCIENCE
CRYSTAL ENGINEERING
MATERIALS

Volume 78 (2022)

Supporting information for article:

Lattice defects in quinacridone

Dominik Brey, Barbara Scherer and Martin U. Schmidt

Supporting information 1. Cif files of the structural models

The following structures are electronically available as cif files in the supporting material:

- Experimental structure of α^{I}-quinacridone, in the unit cell setting used for the simulation of lattice defects (without lattice defects, before lattice-energy minimisation)
- Twin model 1
- Layer misfit structure

Cif files of all other models are available from the corresponding author upon request.

2. Force field evaluation

Table S1 Evaluation of the force field, using the four polymorphic structures of quinacridone. Dreiding calculations were performed with ESP charges and Ewald summation. Lattice energies are reportred in kJ/mol, relative to the α^{I}-phase.

Phase	α^{I}	α^{II}	β	γ
E_{rel} (Dreiding)	0	1.5333	-2.3407	-6.4931
E_{rel} (DFT-D)	0	-1.0869	-5.2935	-6.5214

Table S2 Evaluation of the force-field: Lattice-energy optimiziation on α^{II}-, β-, and γ-quinacridone without lattice defects.

α^{II}-Phase	Experimental	Dreiding	DFT-D
a / Å	7.0500	6.9106	6.797
b / Å	28.4000	29.4171	28.532
c / Å	3.9000	3.9058	3.699
α / °	90.0000	90.0000	90.000
β / °	110.0000	117.1972	111.909
γ / °	90.0000	90.0000	90.000

V / Å^3	733.767	706.223	682.385

β-Phase	Experimental	Dreiding	DFT-D
a / Å	5.692(1)	5.7373	5.775
b / Å	3.975(1)	3.9482	3.724
c / Å	30.02(4)	30.3105	29.570
α / °	90.00	90.0000	90.000
β / °	96.76(6)	96.8244	96.598
γ / °	90.00	90.0000	90.000
V / Å^3	674.502	681.729	627.539

γ-Phase	Experimental	Dreiding	DFT-D
a / Å	13.697(9)	14.0823	13.407
b / Å	3.881(3)	3.8538	3.747
c / Å	13.4020(10)	13.5430	13.065
α / °	90.00	90.0000	90.000
β / °	100.44(1)	100.6366	98.244
γ / °	90.00	90.0000	90.000
V / Å^3	700.63	722.355	649.551

3. Model with lamellar domains with misoriented molecules

Table S3 Lattice parameters of the model of the lamellar domains with misoriented molecules, and of the undisturbed unit cell.

	Lamellar domain	Unit cell
a / Å	3.8402	3.9241
b / Å	6.8962	6.8938
c/ Å	8 * 15.1071	14.9598
α / °	99.864	98.999
β / °	97.700	100.601
γ / °	115.269	114.976
